# Access to skilled attendant at birth and the coverage of the third dose of diphtheria-tetanus-pertussis vaccine across 14 West African countries – an equity analysis

**DOI:** 10.1186/s12939-020-01204-5

**Published:** 2020-06-01

**Authors:** Jacob Albin Korem Alhassan, Oghenebrume Wariri, Egwu Onuwabuchi, Godwin Mark, Yakubu Kwarshak, Eseoghene Dase

**Affiliations:** 1 Department of Community Health and Epidemiology, College of Medicine, University of Saskatchewan, Saskatoon, Canada; 2 African Population and Health Policy Initiative, Gombe, Nigeria; 3 Medical Research Council (MRC) Unit The Gambia, London School of Hygiene and Tropical Medicine, Fajara, The Gambia; 4 Department of Obstetrics and Gynaecology, Federal Teaching Hospital, Gombe, Nigeria; 5 Department of One Health, The University of Edinburgh, Royal (Dick) School of Veterinary Studies, Edinburgh, Scotland UK; 6 Department of Global Health and Management, Institute of Applied Health Sciences, University of Aberdeen, Aberdeen, Scotland UK

**Keywords:** UHC, Equity, West Africa, DTP3, Skilled attendant at birth

## Abstract

**Background:**

Universal Health Coverage (UHC) remains a critical public health goal that continues to elude many countries of the global south. As countries strive for its attainment, it is important to track progress in various subregions of the world to understand current levels and mechanisms of progress for shared learning. Our aim was to compare multidimensional equity gaps in access to skilled attendant at birth (SAB) and coverage of the third dose of Diphtheria-Tetanus-Pertussis (DTP3) across 14 West African countries.

**Methods:**

The study was a cross sectional comparative analysis that used publicly available, nationally representative health surveys. We extracted data from Demographic and Health Surveys, and Multiple Indicator Cluster Surveys conducted between 2010 and 2017 in Benin, Burkina Faso, Cote d’ Ivoire, The Gambia, Ghana, Guinea, Guinea Bissau, Liberia, Mali, Niger, Nigeria, Senegal, Sierra Leone and Togo. The World Health Organization’s Health Equity Assessment Toolkit (HEAT Plus) software was used to evaluate current levels of intra-country equity in access to SAB and DTP3 coverage across four equity dimensions (maternal education, location of residence, region within a country and family wealth status).

**Results:**

There was a general trend of higher levels of coverage for DTP3 compared to access to SAB in the subregion. Across the various dimensions of equity, more gaps appear to have been closed in the subregion for DTP3 compared to SAB. The analysis revealed that countries such as Sierra Leone, Liberia and Ghana have made substantial progress towards equitable access for the two outcomes compared to others such as Nigeria, Niger and Guinea.

**Conclusion:**

In the race towards UHC, equity should remain a priority and comparative progress should be consistently tracked to enable the sharing of lessons. The West African subregion requires adequate government financing and continued commitment to move toward UHC and close health equity gaps.

## Background

Various Low- and Middle-Income Countries (LMICs) have received varied research attention on their progress towards Universal Health Coverage (UHC) [1–4]. UHC is achieved when all persons have access to needed healthcare services without suffering undue financial hardship [4]. As the push towards UHC intensifies, countries in the West African subregion may require more critical research and public policy attention because their health systems continue to be beset by various challenges. Several health systems in the subregion experienced the devastating effects of World Bank-led Structural Adjustment Policies (SAPs) in the mid 1980s which required countries to cut spending to health and social services, effectively placing such responsibilities on households [5]. More recently, some of the countries in this region have faced challenges such as the Ebola outbreak and multiple armed conflicts with manifold negative consequences for health, especially regarding access to essential services for the most vulnerable groups [6–8]. A useful question is whether subregions such as West Africa are making equitable progress in the push for UHC.

As the race towards UHC continues, several important issues have been raised to interrogate the integrity of UHC and to keep it aligned with the fundamental public health goals from which it stems in the first place. Some have argued, for example, for the explicit inclusion of equity considerations in all three dimensions of the famous UHC cube [9]. In so doing, the focus is simply not on increasing population coverage, service provision and financial risk protection but specifying what segments of the population benefit from improvements in the three dimensions [9]. Other authors have argued the need to avoid certain trade-offs in the move towards UHC. For example, on the path to UHC, countries should continue to emphasize equity by ensuring that priority services are covered first [10]. Furthermore, there is the need to focus on low coverage groups while also ensuring that there is a move away from out of pocket payments- which are generally regressive [10]. Paying attention to equity in the move towards UHC is important because it is possible to improve the key dimensions of UHC, while unintentionally, actively worsening health inequities within countries.

Core UHC child health indicators such as the third-dose of Diphtheria-Tetanus-Pertussis (DTP3) vaccination coverage remains low in many parts of the world [11]. In the World Health Organization (WHO) Africa region, particularly West Africa, the median DTP3 coverage is lower compared to other parts of the world such as the Western Pacific region based on 2017 coverage estimates [11]. Access to skilled attendant at birth (SAB), a core maternal health indicator is also lower in many countries of West Africa compared to other world regions [12]. Comparing equitable progress in access to SAB and DTP3 coverages across West Africa would be a important step in providing information on current progress and suggesting ways of ensuring that no one is left behind. However, there is limited research assessing multidimensional equity gaps (i.e. disparities in access to essential health services determined by socioeconomic/geographic differences) in coverage of key UHC maternal and child health indicators across a multi-country context, especially in West Africa.

This paper, therefore, aims to compare multidimensional equity gaps in access to SAB and DTP3 coverage across 14 West African countries as a marker of progress towards UHC. We chose SAB and DTP3 coverage for this analysis for several reasons. Firstly, data availability; the most comparable (and in some cases up to date) data is available for these two specific indicators across the 14 West African countries included in this analysis. Secondly, DTP3 coverage receives huge support from international agencies including the WHO across countries in West Africa, thus, requiring no fee for service (in most cases) at point of access. On the other hand, access to SAB depends, to a large extent, on how a country’s health system is organized and financed, with most countries charging user fees which could exclude the poor. Therefore, examining equity gaps between the two outcome measures might offer further insight on the comparative effectiveness of country and or international level efforts on reducing equity gaps in the push towards UHC.

## Materials and methods

### Study setting and context

We included 14 West African countries (Benin, Burkina Faso, Cote d’ Ivoire, The Gambia, Ghana, Guinea, Guinea Bissau, Liberia, Mali, Niger, Nigeria, Senegal, Sierra Leone and Togo) that had recent (2010 or beyond) disaggregated national health survey data in this analysis. Carbo Verde, although a West African country was not included in the analysis because the latest available data point was for 2005 (which did not meet our definition of ‘recent’). The countries included in our analysis have cultural and geopolitical ties, and shared economic interests and are part of the subregional alliance, the Economic Community of West African States (ECOWAS) [13]. ECOWAS is aimed at promoting subregional integration across several fields, including cross-national health systems collaboration among member states [13]. In 2017, the combined estimated population of the 14 countries was about 372 million, with an estimated 81 million women of reproductive age (15–49 years), and an estimated total surviving infant population of 13 million [14]. Côte d’Ivoire, Ghana, Nigeria, and Senegal were classified as lower-middle income countries based on the 2017 World Bank fiscal year classification, while the others were classified as low income countries.

### Study design

This was a descriptive cross-sectional comparative analysis of current progress in access to SAB and DTP3 coverage, unpacked across four equity dimensions per country in the West Africa subregion. DTP3 coverage is a child health indicator and is one of the sixteen recommended UHC tracer indicators for tracking country level progress towards UHC [15]. The other indicator used in this study, SAB, although not currently a recommended UHC tracer indicator is considered a key indicator of maternal health. We used SAB instead of the current UHC maternal health indicator; *antenatal care visits* (ANC) because most countries in West Africa did not disaggregate ANC by number of visits or by the four dimensions of equity which was the focus of this study. Furthermore, comparable and disaggregated data for SAB for the period under review was available for all countries.

#### Data source

We searched the UNICEF-supported Multiple Indicator Cluster Survey (MICS) and the USAID-supported Demographic and Health Survey (DHS) databases for the most current final report of either household survey for each included country as of June 31, 2019 [16, 17]. The DHS and MICS are large-scale, nationally representative, standardised household surveys. The surveys collect and report health data including access to SAB and DTP3 coverage disaggregated by socioeconomic determinants of inequality including; wealth status, maternal level of education, location of residence, and administrative regions within a country [18]. Their methodologies are considered similar, which may allow for direct comparisons of their data. The methodologies of DHS and MICS are described in detail elsewhere [18]. According to these surveys, SAB is any health professional (including doctor, nurse or midwife) able to provide basic and emergency care to mothers and their newborns during delivery and the postpartum period [16, 17].

### Measuring inequalities in maternal and child health UHC indicators

To evaluate equity gaps in women’s access to SAB and childhood DTP3 immunisation coverage across the 14 West African countries, we stratified both tracer indicators by socioeconomic and geographic determinants of inequality. These include: maternal educational attainment, family wealth status (quintile), location of residence (urban or rural), and regions within a country. The WHO and World Bank 2017 global monitoring report on progress towards UHC recommends these as key dimensions of inequality and should be included in any equity analysis because national averages can mask unequal access to essential services in the most disadvantaged sub-populations [15].

#### Data analysis

The data generated after abstraction from the various data sources were entered into Microsoft Excel® ((Microsoft, Seattle, USA). The Microsoft Excel workbook was formatted according to the WHO Health Equity Assessment Toolkit Plus (HEAT plus) template and imported into the HEAT Plus programme for descriptive analysis and generation of ‘equiplots’ (a plot of equity analysis). HEAT Plus is a software application developed by the WHO to facilitate the assessment of within-country health equity gaps [19]. In addition, HEAT Plus provides a platform for multi-country equity comparison of health outcomes. It uses data to compare health outcomes or coverage of essential services across the different equity dimensions and it is a useful tool for measuring and monitoring inequality [19].

Using this toolkit, the most recent situation (based on the latest available national survey data) of intra-country equity gaps in access to SAB and DTP3 coverage was estimated. We compared coverage between the extremes within each dimension of inequality as a proxy for absolute inequality (i.e. between the most advantaged and the most disadvantaged). Specifically, for family wealth status, we compared coverage in the richest and poorest quintile; for maternal educational attainment, we compared those with at least secondary education and those with no formal education; for region of residence, we compared coverage in the best performing versus that in the worst region within a country; and for place of residence we compared urban versus rural. These within-country analyses were performed for access to SAB and DTP3 immunisation coverage. Finally, to compare progress across the 14 countries, the analyses included inter-country equity comparisons, i.e. equity gaps per country were ranked across the two indicators (access to SAB and DTP3 coverage) and across the four dimensions of equity assessed.

## Results

### Access to SAB in West Africa

**Fig. 1:**
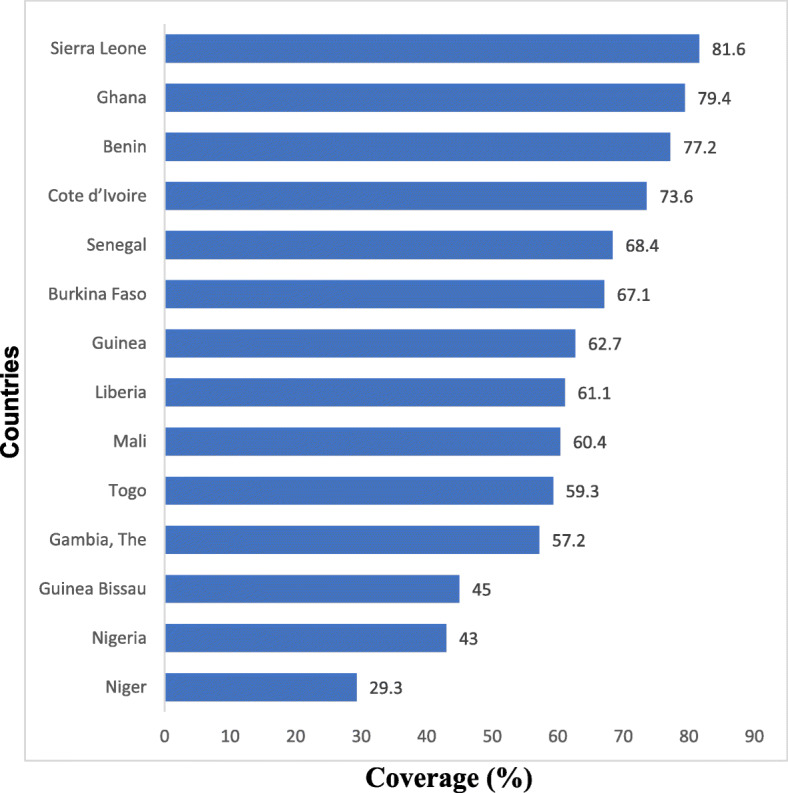
Comparing overall national coverage in access to Skilled Attendant at Birth across the 14 West African Countries (2010–2017). Note: The latest available nationally representative Demographic and Health Survey and Multiple Indicator Cluster Survey data used per country: Benin (MICS 2014), Burkina Faso (DHS 2010), Cote d’Ivoire (MICS 2016), Gambia (DHS 2013), Ghana (MICS 2017), Guinea (MICS 2016), Guinea Bissau (MICS 2014), Liberia (DHS 2013), Mali(MICS 2015), Niger (DHS 2012), Nigeria (MICS 2016/17), Senegal (DHS 2017), Sierra Leone (MICS 2017), Togo (DHS 2013/14)

The overall national coverage in access to SAB ranged between 29.3% in Niger republic to 81.6% in Sierra Leone. Coverage was above 70.0% in Côte d’Ivoire, Benin, Ghana and Sierra Leone. The lowest national coverage in access to SAB were in Niger, Nigeria and Guinea Bissau which each had coverage below 50.0% (Fig. 1).

#### Inequalities in access to SAB across West Africa

**Fig. 2:**
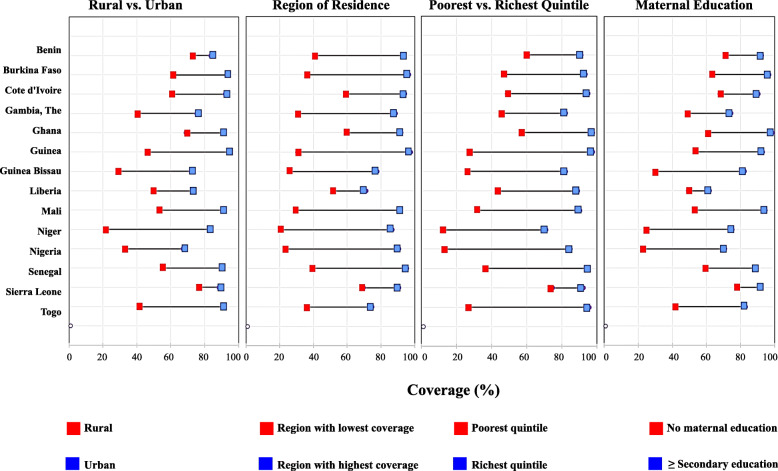
Comparative multi-dimensional equity gaps in access to Skilled Attendant at Birth in the *most disadvantaged* compared to the *most advantaged* women per country across West Africa (2010–2017). Note: The latest available nationally representative Demographic and Health Survey and Multiple Indicator Cluster Survey data used per country: Benin (MICS 2014), Burkina Faso (DHS 2010), Cote d’Ivoire (MICS 2016), Gambia (DHS 2013), Ghana (MICS 2017), Guinea (MICS 2016), Guinea Bissau (MICS 2014), Liberia (DHS 2013), Mali(MICS 2015), Niger (DHS 2012), Nigeria (MICS 2016/17), Senegal (DHS 2017), Sierra Leone (MICS 2017), Togo (DHS 2013/14)

Rural - urban variations in SAB in the West Africa subregion were common, with coverage as low as 21.2–29.3% among people living in rural parts of countries such as Niger and Guinea Bissau respectively. The widest equity gaps in access to SAB for rural versus urban dwellers were in Niger, Togo, Guinea and Guinea Bissau where the percentage difference (percent points) ranged between 42.7–61.8%. In countries such as Benin, Sierra Leone and Ghana, however, the gaps were much smaller ranging from 10.9–21.7% points (Table 1 and Fig. 2).

**Table 1:** Ranking equity gaps in percentage points difference (% points) in access to SAB and DTP3 coverage between the most advantaged and most disadvantaged sub-populations across four equity dimensions in 14 West African countries^**a**^

Skilled Birth Attendance						DTP3 Coverage					
	Rural-Urban	Maternal Education	Wealth Quintile	Regional Difference	Median	Rural- Urban	Maternal Education	Wealth Quintile	Regional Difference	Median	Grand Median
Benin (MICS 2014)	10.9 (1st)	20.9 (3rd)	30.3 (2nd)	53 (7th)	25.6	3.9 (6th)	25 (11th)	29 (10th)	23 (6th)	24	24.5 (6th)
Burkina Faso (DHS 2010)	32.1 (6th)	33.8 (7th)	46.7 (7th)	60.7 (10th)	40.25	2.4 (5th)	4.8 (2nd)	9.5 (6th)	5.7 (1st)	5.25	20.8 (4th)
Côte d’Ivoire (MICS 2016)	30.9 (5th)	22.6 (4th)	46.3 (6th)	35 (4th)	32.95	8.8 (10th)	21.7 (9th)	30 (11th)	27.6 (8th)	24.65	28.8 (7th)
Gambia (DHS 2013)	34.7 (9th)	25.9 (5th)	36.7 (3rd)	57.8 (9th)	35.7	−6.9 (2nd)	−4.7 (1st)	−7.3 (1st)	20.4 (5th)	−5.8	23.15 (5th)
Ghana (MICS 2017)	21.7 (3rd)	37.5 (8th)	40.6 (4th)	32.8 (3rd)	35.15	−0.7 (4th)	7.6 (3rd)	4.5 (3rd)	16 (3rd)	6.05	18.85 (2nd)
Guinea MICS 2016)	48 (12th)	39.3 (9th)	70.5 (13th)	67.1 (13th)	57.55	23.1 (13th)	33.5(13th)	45.7 (13th)	43.3 (12th)	38.4	44.5 (13th)
Guinea Bissau (MICS 2014)	42.7 (11th)	52.6 (14th)	56.7 (8th)	52.5 (6th)	52.55	7.4 (9th)	15.5 (8th)	15.9 (7th)	26 (7th)	15.7	34.35 (10th)
Liberia (DHS 2013)	23.1 (4th)	12.5 (1st)	45.8 (5th)	19.9 (1st)	21.5	−9.1 (1st)	13.6 (7th)	21.5 (8th)	29.5 (9th)	17.55	20.7 (3rd)
Mali (MICS 2015)	38.2 (10th)	41.6 (10th)	58.6 (10th)	61.8 (11th)	50.1	15.1 (11th)	27.3 (12th)	36.4 (12th)	59.5 (14th)	31.85	39.9 (11th)
Niger (DHS 2012)	61.8 (14th)	50.7 (13th)	59.2 (11th)	66.7 (12th)	60.5	21.4 (12th)	24.7(10th)	23.6 (9th)	34.8 (10th)	24.15	42.75 (12th)
Nigeria (MICS 2016/17)	34.2 (7th)	48.5 (12th)	72.1 (14th)	67.7 (14th)	58.1	25.5 (14th)	64.9(14th)	53.1 (14th)	52.7 (13th)	52.9	53 (14th)
Senegal (DHS 2017)	34.4 (8th)	30.3 (6th)	58 (9th)	56 (8th)	45.2	6.2 (8th)	7.9 (4th)	1.9 (2nd)	38.6 (11th)	7.05	32.35 (9th)
Sierra Leone (MICS 2017)	11.2 (2nd)	14.8 (2nd)	17.4 (1st)	21 (2nd)	16.1	−0.8 (3rd)	8.4 (5th)	6.5 (4th)	13.1 (2nd)	7.45	12.15 (1st)
Togo (DHS 2013/14)	50.4 (13th)	41.6 (11th)	68.7 (12th)	39 (5th)	46	4.3 (7th)	13 (6th)	6.9(5th)	19.9 (4th)	9.95	29.45 (8th)

^a^Each figure represents the difference between two extremes: Rural-Urban (coverage in urban-rural); Maternal education (coverage in mothers with maternal education of secondary plus-mothers with no education); Wealth quintile (coverage in women or children in the richest quintile- women in the poorest quintile); Regional difference (coverage in best performing region-worst performing region)

Source: Authors, based on MICS (Multiple Indicator Cluster Survey) and DHS (Demographic and Health Survey)

The widest within-country regional differences (region with highest access compared to region with lowest access) in SAB were in Nigeria, Niger and Guinea while the narrowest regional equity gaps in access to SAB were in Liberia, Sierra Leone and Ghana (Fig. 2).

Using family wealth status, the widest equity gap in access to SAB was in Nigeria, followed by Guinea, Togo, Niger and Mali. In Nigeria, there was almost a 72.0% point gap in access to SAB between women in the wealthiest compared to those in the poorest quintile (84.9% vs 12.8%). The countries with narrowest gaps were Sierra Leone followed by Benin, Gambia and Ghana where the percentage difference by wealth quintile were between 17% and 40% points (Fig. 2).

Disaggregating SAB by maternal education, the widest equity gap between the extremes compared was observed in Guinea Bissau (52.6% points), Niger (50.7% points) and Nigeria (48.5% points) (Table 1 and Fig. 2). The narrowest equity gap was in Liberia, followed by Sierra Leone, Benin and Côte d’Ivoire where the gaps in coverage between educated and non-educated mothers was less than 30.0% in each case. Overall, equity gaps were most evident when access to SAB was stratified by region, followed by differences in wealth quintile, then maternal education and the narrowest was due to rural-urban differences in access to SAB.

### DTP3 coverage across West Africa

**Fig. 3:**
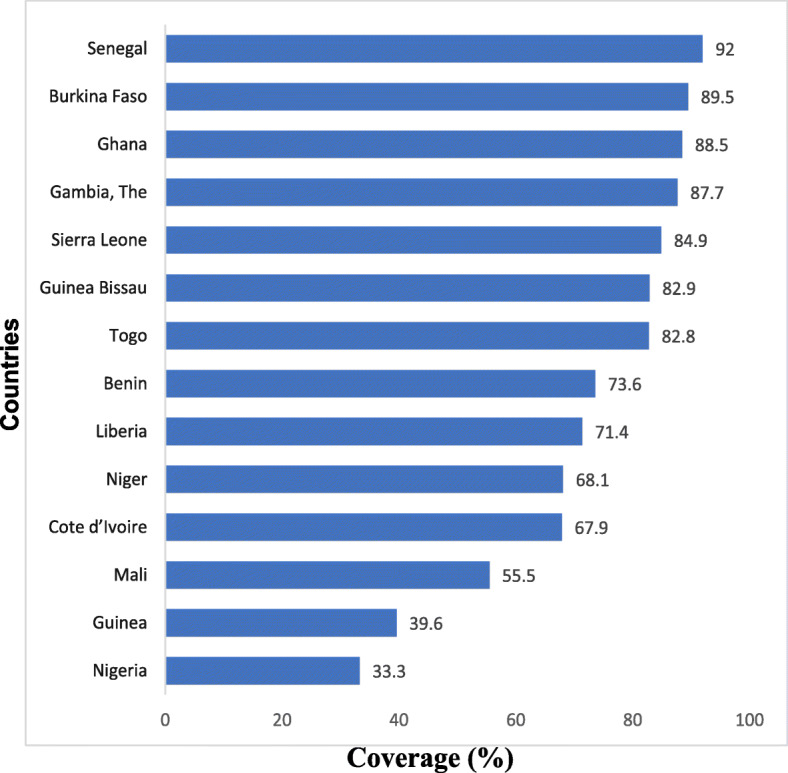
Comparing overall national Diphtheria-Tetanus-Pertussis (DTP3) containing vaccine coverage across the 14 West African Countries (2010–2017). Note: The latest available nationally representative Demographic and Health Survey and Multiple Indicator Cluster Surveys data used per country: Benin (MICS 2014), Burkina Faso (DHS 2010), Cote d’Ivoire (MICS 2016), Gambia (DHS 2013), Ghana (MICS 2017), Guinea (MICS 2016), Guinea Bissau (MICS 2014), Liberia (DHS 2013), Mali(MICS 2015), Niger (DHS 2012), Nigeria (MICS 2016/17), Senegal (DHS 2017), Sierra Leone (MICS 2017), Togo (DHS 2013/14)

The overall national DTP3 coverage ranged between 33.3% in Nigeria to 92.0% in Senegal. It was above 85.0% in Gambia, Ghana, Burkina Faso and Senegal. The lowest national DTP3 coverage was in Nigeria and Guinea which had coverage of below 40.0% of the population of surviving infants (Fig. 3).

#### Inequalities in DTP3 coverage across West Africa

**Fig. 4:**
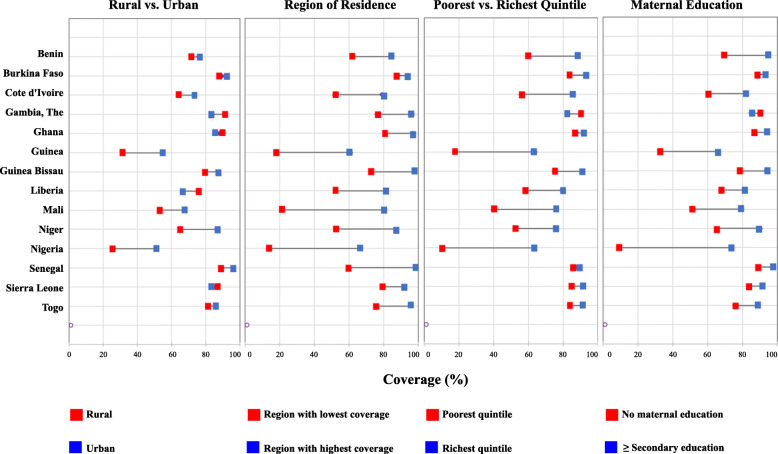
Comparative multi-dimensional equity gaps in Diphtheria Tetanus and Pertussis (DTP3) containing vaccine coverage in the *most disadvantaged* compared to the *‘most advantaged’* children per country across West Africa (2010–2017). Note: The latest available nationally representative Demographic and Health Survey and Multiple Indicator Cluster Surveys data used per country: Benin (MICS 2014), Burkina Faso (DHS 2010), Cote d’Ivoire (MICS 2016), Gambia (DHS 2013), Ghana (MICS 2017), Guinea (MICS 2016), Guinea Bissau (MICS 2014), Liberia (DHS 2013), Mali(MICS 2015), Niger (DHS 2012), Nigeria (MICS 2016/17), Senegal (DHS 2017), Sierra Leone (MICS 2017), Togo (DHS 2013/14)

Overall, equity gaps in DTP3 coverage were narrower compared to the gaps in SAB described above. Indeed, in Liberia and the Gambia, DTP3 coverage among children living in rural settings was higher than for those living in urban settings. In many instances, the countries with the widest equity gaps in SAB were the same countries with the widest equity gaps in DTP3 coverage. The equity gap in DTP3 coverage between children in rural and urban settings for countries like Nigeria, Guinea and Niger were the widest across the subregion. Comparing intra -country inequities in DTP3 coverage, the widest equity gaps were in Mali followed by Nigeria and then Guinea. These same countries also had lowest overall DTP3 coverage in the subregion. Burkina Faso, Sierra Leone and Ghana had the narrowest regional equity gaps (Fig. 4).

The widest wealth-based equity gaps in DTP3 coverage were in Nigeria followed by Guinea and Mali. In Nigeria where the equity gap in DTP3 coverage by wealth quintile was the largest, people in the poorest wealth quintile had DTP3 coverage levels as low as 10.2% and the gap between the richest and the poorest was as wide as 53.1% points. Mothers in the poorest quintile in Guinea had DTP3 coverage rates under 20.0% with a gap of about 46% points compared to those in the richest quintile (Table 1 and Fig. 4). The countries with the smallest wealth-based equity gaps in DTP3 coverage were Gambia, Senegal and Ghana where the gap between the wealthiest and the poorest was not more than 10.0% points in each case. Indeed, in the Gambia, DTP3 coverage among those in the poorest wealth quintile was 7.0% more than those in the richest quintile.

Nigeria, Guinea and Mali had the largest gaps in DTP3 coverage by maternal education. The differences in DTP3 coverage between the children of mothers who had no formal education and those whose mothers had at least secondary education stood at 64.9%, 33.5% and 27.3% points for Nigeria, Guinea and Mali respectively. In countries like Burkina Faso, Ghana, Gambia and Senegal, equity gaps were quite narrow, with the difference between DTP3 coverage among children of mothers with at least secondary education and those with no education below 10% points in each case. Similar to SAB, equity gaps in DTP3 coverage were most evident when stratified by region, followed by differences in wealth quintile, then maternal education and the narrowest was due to rural-urban differences in access to DTP3 coverage.

## Discussion

Overall, equity gaps in access to SAB were much wider than the gaps that existed for DTP3 coverage. Nigeria, Niger and Guinea, compared to other countries in West Africa, had the widest equity gaps between the most advantaged compared to the most disadvantaged sub-populations. Conversely, countries such as Benin, Sierra Leone, Liberia and Ghana generally had much smaller equity gaps across indicators.

Our findings agree with those of other authors who have identified Mali, Niger and Nigeria as having large equity gaps in SAB and DTP3 coverage [11, 12, 20]. Indeed, these gaps are similar to the large gaps found in other countries such as Ethiopia, Bangladesh and Haiti outside our study region [12]. In terms of coverage for DTP3, other studies have also found wider equity gaps in coverage in Nigeria and placed it in a similar category with others such as the Democratic Republic of Congo, Ethiopia, Indonesia and Pakistan which have been described as ‘high priority’ countries given their large gaps in equity [21]. These findings underscore the importance of within-country equity analyses for various UHC indicators, using similar and timely data, thus reducing the likelihood of ‘leaving anyone behind’ as countries pursue UHC.

Additionally, our findings raise the question on the comparative effectiveness and reach of vertical (donor/disease-specific) or horizontal (in-country/whole health system) programs. Immunization programs (including DTP3 vaccination) is driven across many West African countries mainly by international donor funding and support from organisations/institutions such as Gavi. There appears to have been an acceleration in progress not only in terms of higher DTP3 coverage rates but also in closing equity gaps in DTP3. On the other hand, services such as SAB which reflect the responsiveness of the underlying health system, are driven mainly by in-country funding and seldom by vertical (donor disease-specific funding) support [22]. Thus, poor in-country financing (horizontal) of services could partly explain the wider multidimensional equity gaps in access to SAB across many West African countries. The response to the question of horizontal or vertical funding may require a two-prong approach to close the identified equity gaps. Firstly, continued government commitment in ensuring that health systems are adequately funded with priority on maternal and child health services. Secondly, a 'diagonal approach' is suggested by some authors where there is a movement beyond the vertical-horizontal polarization of health system funding to a combination where countries can receive diseases-specific funding and the overarching health system also continue to receive support [23].

It is important to emphasize that the solution to health equity gaps described here are not simply the provision of more services but also critical attention to the structural and social determinants of health [24]. Living in a rural area, belonging to the lowest wealth quintile or being born to an uneducated mother should not automatically reduce a child’s chance of receiving vaccination or a woman’s chance to have access to a skilled attendant while having her baby. This research does not aim to unduly castigate countries that have the widest gaps, as the drivers of inequalities can sometimes be factors outside a country. That notwithstanding, governments in the countries with the largest equity gaps need to pay attention not only to the ‘health care system’ but those factors outside this system, that is, in the broader ‘health system’ (i.e. including the non-medical components) which determine overall health and access to needed health services in the first place. In the sections that follow, we examine some factors that may be useful for understanding differential progress in the West African subregion by using the examples of Sierra Leone and Nigeria. We chose both countries based on how wide or narrow their equity gaps were and to provide a picture of both extremes. Compared to other countries, Sierra Leone had relatively narrow multidimensional equity gaps across both indicators. Nigeria, on the other hand, had relatively wide multidimensional equity gaps across both tracer UHC indicators.

### Understanding the ‘miracle’ of Sierra Leone

Based on our analysis, Sierra Leone can be considered as an ‘outlier’ highly successful country in the West African subregion. This is because the differential equity gap between the most and the least advantaged sub-groups in access to SAB and DTP3 coverage across all equity dimensions was substantially less than the recommended 20% points benchmark for monitoring equity [25]. Ordinarily, this finding could be considered a ‘miracle’ because of various health system shocks suffered by Sierra Leone such as the decade-long civil war which ended in 2002, the Ebola outbreak in 2014–2016 and the country’s low-income status.

In a landscape of a weak health system, and in response to high child and maternal mortality figures, the government of Sierra Leone implemented the Free Health Care Initiative (FHCI) in 2010 [26]. The FHCI was not focussed on providing free services alone as has been the norm in some sub-Saharan African contexts. Rather, it recognised that the core health system pillars including medical supplies, health workforce, governance, infrastructure, information, financing, monitoring and evaluation needed reinforcing if its goal was to be achieved [26]. Furthermore, FHCI which was particularly focussed on children and women, involved a ‘whole system’ change, and was implemented across all regions in Sierra Leone simultaneously.

The impact evaluation of the FHCI between 2010 and 2015 revealed that this ambitious reform responded to a clear need, and was well designed to produce the needed changes in a holistic, system-wide approach rather than a programme focused only on user-fee removal [27]. Furthermore, FHCI was seen as an important factor that contributed to improvements in coverage of essential services for mothers and children including access to SAB and DTP3 coverage, and narrowing of equity gaps based on socioeconomic or geographic factors [27]. In a weak health system, like post-conflict Sierra Leone, user-fee removal in addition to structural changes to the health system were key drivers of progress towards UHC in terms of coverage and equity because the majority of the population lived below the poverty line. Similar policies like the FHCI have been adopted in Ghana where proceeds from VAT has been used to prioritise free health care for children under-5 and mothers under the National Health Insurance Scheme (NHIS). This policy shift and a focus on women and children similar to the scenario in Sierra Leone may have also accounted for the narrow equity gaps in access to SAB and DTP3 coverage across the dimensions of equity assessed in Ghana. In the race toward UHC, governments of other relatively poorly performing countries could adopt these useful local solutions which could be implemented with sensitivity to local and contextual differences.

### Interrogating Nigeria’s wide equity gaps

Despite the country’s oil wealth, Nigeria, consistently had the widest gaps between the most and least advantaged sub-populations across most equity dimensions for both UHC indicators assessed. Furthermore, coverage at the most favourable ends of the equity dimensions in access to SAB and DTP3 coverage in Nigeria is lower than the least favourable ends in other West African countries. Several factors including poor government financing of health, unavailability of services or imposition of user fees which exclude the poorest and most vulnerable sub-populations could explain the low overall coverage and persistently wide equity gaps in access to SAB and DTP3 coverage in Nigeria [26, 27]. These issues may not be peculiar to Nigeria as other countries in West Africa continue to grapple with similar challenges. However, government’s efforts aimed at remedying the situation and pushing Nigeria towards UHC has been sub-optimal over the past two decades.

The Government of Nigeria in 1999 launched the NHIS, a social health insurance scheme as a pathway to achieving UHC and removing high out-of-pocket user fees, however, not until 2005 did it become fully operational [28]. After almost two decades of its implementation, only 4% of about 200 million Nigerians have been covered by the scheme, with the majority being formal sector federal government workers, while the most vulnerable people who work in the informal sector continue to be excluded [29]. Furthermore, additional issues of poor financing, and non-adoption of the NHIS scheme by sub-National governments due to its non-mandatory nature has also been shown to be responsible for the poor progress of the scheme [29]. For example, Nigeria’s expenditure on health as a percentage of total national budget was only 4% in 2018. The highest over the past two decades was the 6% of the national budget spent on health in 2012, meaning that expenditure on health as a proportion of the national budget in Nigeria consistently lags far behind the 15% recommended by the Abuja declaration [30]. The factors above explain, at least, in part the wide multi-dimensional equity gaps between the most advantaged (who often can afford out-of-pocket user fees) and the most disadvantaged sub-populations across both UHC indicators assessed in Nigeria.

Central to UHC is bridging the equity gap so that the most vulnerable in society are not left behind. Attainment of equitable access to needed health services requires increased political will/action, and huge fiscal investment in Nigeria to ensure that the most vulnerable sub-populations including non-formal sector workers, rural dwellers, people without formal education, and the poorest are not excluded from the needed health services.

### Limitations

There are inherent limitations to our analysis which should be considered. Firstly, the timepoints for the different data sets used are not always comparable. The oldest data in our analysis was from 2010 and the latest was from 2017. Thus, although Burkina Faso, Benin, Gambia or Liberia appear to be performing very well across most equity indicators it is possible that the most up-to-date data when collected may show these countries to be performing poorly (or much better than is seen in this analysis). For the countries with poorer performance such as Togo and Niger, it is also possible that more recent data may show substantial improvements (or worsening of equity gaps). That notwithstanding, many countries included in our analysis (which have excellent performance such as Sierra Leone and Ghana or poor performance like Guinea and Nigeria) have very recent (2017) data. Thus, while time can influence our analysis, there are still noteworthy variations in equity performance for countries with the most recent and comparable data.

Secondly, our analysis used data from both the DHS and MICS. While both are nationally representative and similar in several respects, they are still different datasets collected by different organizations for different purposes. This limitation may not have significantly influenced our analysis because we relied on equity dimensions within the given countries and are restricted to the same data set (MICS or DHS). Finally, the data collection approaches used in both surveys relied on maternal recall of childhood immunization history [18]. Although this approach increases the availability of immunization coverage data globally, especially in low-resources settings, it can sometimes be subject to maternal recall bias. While we acknowledge the limitations of DHS and MICS, they represent the best available population-based and nationally representative datasets, thus their findings are useful for informing health policies.

## Conclusion

UHC is an attainable goal but requires significant global, regional and national commitment with critical attention to equity so that the most vulnerable are not left behind. As seen in the case of countries such as Sierra Leone, the implementation of programs such as FHCI whose core aim was to promote UHC can play a big role in improving health services access and closing gaps in health equity. As the journey continues towards UHC, more comparative research needs to be conducted periodically to monitor and track progress and importantly to explain progress and the mechanisms responsible for it. This would offer more insight to countries falling behind.
